# Multi-Voxel Decoding and the Topography of Maintained Information During Visual Working Memory

**DOI:** 10.3389/fnsys.2016.00002

**Published:** 2016-02-15

**Authors:** Sue-Hyun Lee, Chris I. Baker

**Affiliations:** ^1^Department of Bio and Brain Engineering, College of Engineering, Korea Advanced Institute of Science and Technology (KAIST)Daejeon, South Korea; ^2^Laboratory of Brain and Cognition, National Institute of Mental Health, National Institutes of HealthBethesda, MD, USA

**Keywords:** working memory, short term memory, multivoxel pattern analysis (MVPA), visual imagery, visual working memory, fMRI

## Abstract

The ability to maintain representations in the absence of external sensory stimulation, such as in working memory, is critical for guiding human behavior. Human functional brain imaging studies suggest that visual working memory can recruit a network of brain regions from visual to parietal to prefrontal cortex. In this review, we focus on the maintenance of representations during visual working memory and discuss factors determining the topography of those representations. In particular, we review recent studies employing multi-voxel pattern analysis (MVPA) that demonstrate decoding of the maintained content in visual cortex, providing support for a “sensory recruitment” model of visual working memory. However, there is some evidence that maintained content can also be decoded in areas outside of visual cortex, including parietal and frontal cortex. We suggest that the ability to maintain representations during working memory is a general property of cortex, not restricted to specific areas, and argue that it is important to consider the nature of the information that must be maintained. Such information-content is critically determined by the task and the recruitment of specific regions during visual working memory will be both task- and stimulus-dependent. Thus, the common finding of maintained information in visual, but not parietal or prefrontal, cortex may be more of a reflection of the need to maintain specific types of visual information and not of a privileged role of visual cortex in maintenance.

## Introduction

Working memory commonly refers to our ability to maintain and manipulate stimulus representations, typically for a short period of time, in the absence of the ongoing presence of that stimulus (Baddeley and Hitch, 1974). For example, holding a phone number in mind prior to pressing the buttons on the phone. In vision, working memory can involve diverse types of maintained content from complex forms such as faces and objects to fine visual details such as specific orientations or colors. The neural basis of visual working memory has long been the subject of debate and while multiple brain areas, from visual cortex, including primary visual cortex (V1) and the middle temporal area (MT), to the parietal, temporal and prefrontal cortices have been implicated in visual working memory (Wager and Smith, 2003), the functional roles these regions play has been controversial. Typically, theories have distinguished different processes that might be involved in visual working memory (Eriksson et al., 2015), making a distinction between stimulus representation or storage and executive or top down control, and have tried to map those distinctions onto specific brain regions. Various accounts posit that there is a working memory system separate from other memory or perception systems (e.g., Baddeley, 2012), that prefrontal cortex is involved in both maintenance and executive control (e.g., Funahashi et al., 1989, 1993; Chafee and Goldman-Rakic, 1998; Constantinidis et al., 2001), or that information is maintained in posterior cortex with prefrontal cortex primarily involved in top-down control of those regions (for recent review, see D’Esposito and Postle, 2015). In this review, we will focus on recent evidence from human functional magnetic resonance imaging (fMRI) studies identifying the substrates of maintained representations during visual working memory.

The terms “visual working memory” and “visual short-term memory” are often used interchangeably. One of the key components of working memory is indeed the short-term maintenance of visual representations. However, working memory is often used to describe not just maintenance of representations, but internal manipulation of those representations as well (for recent discussion, see Marois, 2015; Postle, 2015a). In this review, we will refer to “visual working memory”, following many of the studies that we cite, although our primary focus is on the maintenance of visual representations. Such maintenance can occur in many different contexts. For example, a participant might be asked to remember a stimulus that is briefly flashed on the screen (e.g., Serences et al., 2009). Alternatively, a participant might be cued to recall a recently presented stimulus, out of two or more alternatives, and then asked to remember that stimulus over a delay period (e.g., Harrison and Tong, 2009). However, the representations that are being maintained need not be accessed from recent sensory experience, but can also be retrieved from long-term memory, allowing further manipulation of the remembered content in such a way that makes it useful for ongoing behavior. In this light, visual working memory may share mechanisms with visual imagery (Albers et al., 2013; Tong, 2013) and even the accessing of conceptual knowledge (Martin, 2007, 2015).

In this review, we will highlight that to understand the engagement of particular regions during working memory, it is important to consider the nature of the stimulus representations that are being maintained. We will use the term “information” to refer to the specific aspects of the presented stimulus that are relevant to task performance and must therefore be remembered over the delay period. Thus, “information” does not necessarily refer to the entire stimulus itself or even to sensory properties of the stimulus. The maintained information could be one aspect of a visually presented stimulus (e.g., color, but not orientation, of a grating stimulus), or an abstraction from the stimulus (e.g., category). Further, the same information could be contained in very different underlying representations. For example, stimulus position could be maintained either in a visual representation (e.g., in V1) or a motor representation for an upcoming eye movement.

The fMRI studies we focus on have employed multivoxel pattern analysis (MVPA) techniques to decode maintained representations during the delay periods of working memory tasks. By “decoding” we simply mean that the BOLD response measured with fMRI has been used to infer the information that is represented. Many of these studies have revealed maintained representations in visual cortex (e.g., V1-V4, MT), supporting a role of sensory, not prefrontal, cortex in maintenance. However, there is some evidence for maintenance outside of visual cortex (including posterior parietal and prefrontal cortex) and here, we suggest that the ability to maintain information is a general property of cortex, not limited to specific regions. We argue that the predominance of studies revealing maintained representations in early visual cortex reflects the stimuli and task that have been probed. Specifically, the recruitment of any region will reflect the particular information that must be maintained as determined by the task context and the behavioral goals. Thus, working memory is best understood as a highly distributed process wherein information can be maintained in any systems engaged in the initial perceptual processing. This includes not just sensory cortex, but any region contributing to the initial percept, including parietal and frontal areas.

## Decoding Maintained Representations

The notion that information is maintained in sensory regions during visual working memory has been referred to as the “sensory recruitment” hypothesis (Pasternak and Greenlee, 2005). Early support for this view came from perceptual discrimination studies in which participants had to detect whether a sample stimulus (varying in spatial frequency, orientation, or motion stimulus) matched a test stimulus presented after a brief delay (Dupont et al., 1998; Magnussen and Greenlee, 1999). Irrelevant stimuli presented during the delay were found to interfere with discrimination performance in a feature-selective manner, suggesting that the mechanisms involved in maintaining the representation of the sample stimulus are linked to those involved in perceptual processing (Magnussen et al., 1991; Magnussen and Greenlee, 1992).

However, physiology (e.g., Funahashi et al., 1989, 1993; Miller et al., 1996; Constantinidis et al., 2001) and early fMRI (e.g., Zarahn et al., 1997; Courtney et al., 1998; Jha and McCarthy, 2000; Leung et al., 2002) studies shifted the emphasis away from sensory cortex to prefrontal cortex with the observation of elevated activity during the delay period that spanned intervening stimuli. While it was appealing to equate maintained activity with maintained representations, the mere presence of elevated activity does not indicate the nature of the underlying processing (Curtis and D’Esposito, 2003; Sreenivasan et al., 2014a). Further, such increased activity can also be found in posterior brain areas (Ranganath and D’Esposito, 2005) for both simple (Greenlee et al., 2000) and complex (Courtney et al., 1997; Druzgal and D’Esposito, 2003; Ranganath et al., 2004; Oh and Leung, 2010) visual features.

An alternative approach, focusing on the capacity limit of working memory, highlighted the potential role of parietal cortex. In particular, regions in parietal cortex exhibit activity which tracks the number of items held in memory and correlates with apparent capacity limitations (Linden et al., 2003; Todd and Marois, 2004, 2005; Vogel and Machizawa, 2004; Xu and Chun, 2006; Harrison et al., 2010). Further, Mitchell and Cusack (2008) found correlation with capacity-based regressors not only in parietal cortex but also in some prefrontal areas. While these findings suggest a link between parietal (and possibly prefrontal) cortex and working memory capacity, they do not indicate that the representations are maintained in these regions.

Recent fMRI studies have now provided more compelling evidence for the sensory recruitment model by focusing on whether the responses in a given region are specific to the maintained information (D’Esposito and Postle, 2015). Such studies have taken advantage of the development of MVPA techniques (for reviews, see Norman et al., 2006; Serences and Saproo, 2012; Haynes, 2015), which focus on the patterns of response across voxels rather than the average magnitude (see Table 1 for a summary of studies). In these studies, the BOLD responses in a given region are used to infer or “decode” the nature of the underlying representation. For example, Harrison and Tong (2009) presented participants with two serially presented gratings, followed by a retro-cue (“1” or a “2”) indicating whether they had to remember the first or second grating. A test grating was presented after a further delay of 11 s and participants had to indicate whether it was rotated clockwise or anticlockwise relative to the cued grating. There were three key findings. First, during the delay period, the patterns of BOLD response in early visual cortex (V1-V4) could be used to decode the orientation of the grating held in memory, suggesting that early visual cortex holds a specific representation of the maintained orientation. Second, this decoding was possible even when there was no elevated activity during the delay period, suggesting that elevated activity is not necessary for the maintenance of orientation information. Third, the patterns of response observed during the delay period were similar to those evoked by physically presented gratings, suggesting that the maintained representations are strongly related to perceptual representations in these areas.

**Table 1:** **Summary of studies demonstrating multi-voxel decoding of information during visual working memory**.

Reference	Stimuli	Task-relevant information	Information decoded	Cortical regions allowing decoding
Ester et al. (2009)	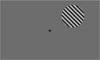	Orientation	Orientation	V1
Harrison and Tong (2009)	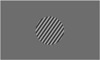	Orientation	Orientation	V1-V4
Serences et al. (2009)	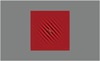	a) Orientation b) Color	a) Orientation b) Color	a) V1 b) V1
Christophel et al. (2012)	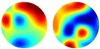	Color pattern features	Color pattern identity	Early visual Posterior parietal
Jerde et al. (2012)	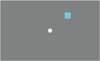	Stimulus position	Left vs. right visual field	IPS2, IPS3 PCS
Linden et al. (2012)	Faces, Bodies, Scenes, Flowers	Exemplar identity	Category	Early visual Parahippocampal
Riggall and Postle (2012)	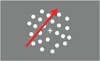	a) Direction b) Speed	a) Direction b) None	a) Lateral occipital and temporal Medial occipital b) None
Sneve et al. (2012)	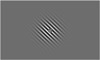	a) Orientation b) Spatial frequency	a) Orientation b) Spatial frequency	a) V1-V4, LO1 b) V1, V2, V3A/B
Albers et al. (2013)	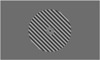	Orientation	Orientation	Superior frontal gyrus Supramarginal gyrus V1-V3
Emrich et al. (2013)	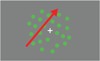	Direction (cued by color)	Direction	Intraoccipital sulcus MT+ V1, V2
Ester et al. (2013)	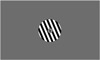	Orientation	Orientation	V1, V2
Han et al. (2013)	Faces Scenes	Exemplar identity	Category	Face-selective (FFA, OFA) Scene-selective (PPA, TOS, RSC)
Lee et al. (2013)	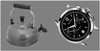	a) Visual features b) Object name	a) Object identity b) Object identity	a) Posterior fusiform b) Lateral prefrontal
Nelissen et al. (2013)	Bodies, Faces, Houses	Exemplar identity	Category	Body-selective (EBA) Face-selective (FFA) Scene-selective (PPA) Object-selective (LOC)
Xing et al. (2013)	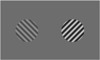	Stimulus contrast	Stimulus contrast	V1, V2
Christophel and Haynes (2014)	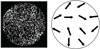	Motion flowfield features	Motion flowfield identity	MT+ Posterior parietal Somatosensory
Naughtin et al. (2014)	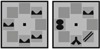	Exemplar identity with location	a) Identity of whole object set b) Number of objects	a) Right dorsolateral prefrontal Premotor Left inferior frontal junction Anterior cingulate Superior medial frontal Left sIPS, ilPS Left LOC b) Left premotor sIPS, ilPS LOC
Pratte and Tong (2014)	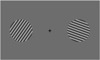	Position-specific orientation	Position-specific orientation	Contralateral V1, V2 Bilateral V3AB, V4
Sprague et al. (2014)	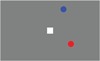	Stimulus position	Stimulus position	V1-V4, V3A IPS0-IPS3 Superior PCS
Sreenivasan et al. (2014b)	Faces Scenes	Exemplar identity	Category	Extrastriate visual cortex Lateral prefrontal cortex
Christophel et al. (2015)	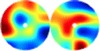	Color pattern features	Color pattern identity	Early visual Posterior parietal
Ester et al. (2015)	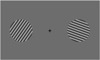	Orientation	Orientation	Bilateral V1, Contralateral V4 Ipsilateral IPS2, IPS3 Prefrontal (incl. PCS)

*Studies are organized first by date and then alphabetically by first author. Across studies, a wide range of visual stimuli have been employed, from oriented gratings to high–level stimuli such as faces, objects and scenes. We list both the task-relevant information as well as the information that could be decoded. In many cases, these are the same, but there are also some studies in which the level of decoding differed from the task-relevant information. For example, in several of the studies employing high-level visual stimuli, the task required maintenance of information about within-category exemplars (e.g., different faces or scenes), but the decoding was at the level of category (e.g., faces vs. scenes). In the final column, we list the major regions in which information could be decoded. Studies differed in how regions were identified (e.g., region-of-interest vs. searchlight analyses) and we adopt the level of description provided in the published studies. We ascribe decoding to particular functional regions (e.g., V1, MT, FFA) only if those regions were specifically localized. Further, note that we do not give any information about tested regions in which information could not be decoded. For this information, we refer readers back to the original cited papers. EBA, Extrastriate Body Area; FFA, Fusiform Face Area; IPS0–4, retinotopically-defined regions in and around the intra-parietal sulcus (iIPS, inferior intra-parietal sulcus; sIPS, superior intra-parietal sulcus); LOC, object-selective Lateral Occipital Complex; LO1, lateral occipital area 1; MT+, motion-selective areas including both the middle temporal (MT) and medial superior temporal (MST) areas; OFA, Occipital Face Area; PCS, precentral sulcus; PPA, Parahippocampal Place Area; RSC, scene-selective Retro Splenial Complex; TOS, scene-selective region near the Transverse Occipital Sulcus; V1-V4, retinotopically defined regions of early visual cortex*.

Support for the maintenance of representations in early visual cortex has also been provided by an alternative approach in which the response properties of individual voxels are explicitly modeled. For example, Ester et al. (2013) fit a model (often termed an encoding model) of orientation selectivity, based on a set of eight orientation-selective response functions “channels”, to each voxel in early visual areas (following the approach of Brouwer and Heeger, 2009, 2011). Then, based on the response pattern across voxels (in independent data), they could reconstruct images reflecting the information content in a given area during the delay period of the task. This analysis revealed graded response profiles in V1 and V2 that peaked for the remembered orientation and was only present when explicit memory was required.

The ability to decode maintained orientation information in early visual cortex during visual working memory has now been replicated multiple times, supporting the three key findings described above (Ester et al., 2009, 2015; Serences et al., 2009; Sneve et al., 2012; Albers et al., 2013; Pratte and Tong, 2014). Further, the precision of the orientation representations in early visual cortex, measured as memory load is varied, reflects behavioral performance (Ester et al., 2013; see also Emrich et al., 2013). Beyond orientation, decoding of maintained representations has also been reported in early visual cortex for contrast (Xing et al., 2013), location (Sprague et al., 2014), motion (Riggall and Postle, 2012; Emrich et al., 2013), color (Serences et al., 2009), and color patterns (Christophel et al., 2012, 2015).

In all of these cases, the information that can be decoded during visual working memory is the kind of information (e.g., orientation, color, contrast) that is well represented by the underlying stimulus feature-selectivity in early visual cortex. Similarly, other areas of visual cortex with more specialized feature-selectivity during perception have demonstrated maintenance of information corresponding to that selectivity. For example, decoding of simple (Riggall and Postle, 2012; Emrich et al., 2013) and complex motion information (Christophel and Haynes, 2014) has been reported in the human MT complex (MT+) that is highly selective for stimulus motion. Further, in studies that have tested working memory for complex images such as objects, scenes and faces, decoding of maintained information has been reported in category-selective occipitotemporal cortex (Linden et al., 2012; Han et al., 2013; Lee et al., 2013; Nelissen et al., 2013; Sreenivasan et al., 2014b). However, it is important to note that in many of these cases while the task required within-category information (e.g., individual faces or scenes), decoding was at the level of category (e.g., faces vs. scenes, see Table 1). Thus, the ability to maintain representations appears to be a general property of visual cortex, with regions maintaining representations of those stimuli that match their underlying stimulus-selectivity.

It is important to realize, however, that the maintenance of content during delay periods is not simply a passive reflection of stimulus properties. The nature of the information maintained is critically dependent on the task, which determines the specific information that is required for successful performance. For example, Serences et al. (2009) presented colored oriented gratings and varied whether color or orientation was relevant for the discrimination to be made after the delay. They found that both orientation and color could be decoded from V1 during the delay, but only when that specific feature information was task-relevant. Similarly, while there is some evidence that orientation information is maintained throughout V1, not just in the part of the retinotopic map corresponding to the stimulus location in the visual field (Ester et al., 2009, 2015), location-specific orientation information can be decoded when both location and orientation are task-relevant (Pratte and Tong, 2014). Consistent with this, Lee et al. (2013) reported decoding of object identity in high-level visual cortex only when the visual properties of the presented stimuli were task-relevant.

In contrast to the ability to decode maintained information in visual cortex during working memory, studies investigating parietal and frontal cortex have often failed to find any evidence for maintained representations. For example, while Riggall and Postle (2012) could decode maintained information about motion direction in early visual cortex and MT+, this was not possible in frontal and parietal areas, even when selecting those areas that showed elevated activity during the delay. Similarly, Emrich et al. (2013) found that the ability to decode multiple items in memory decreased significantly with increasing load in early visual cortex and MT+, but could not decode remembered items in parietal cortex, even in those areas that showed load-sensitive delay period activity. These results argue strongly for the sensory recruitment model and suggest that neither elevated nor load-sensitive delay activity is a sufficient marker for maintained representations in working memory.

However, these failures to find evidence for maintained representations outside visual cortex should be treated cautiously since some studies have reported positive results (Christophel et al., 2012, 2015; Jerde et al., 2012; Lewis-Peacock and Postle, 2012; Han et al., 2013; Christophel and Haynes, 2014; Naughtin et al., 2014; Sprague et al., 2014; Ester et al., 2015). For example, in studies of working memory for colored patterns and motion flow patterns, Christophel and colleagues (Christophel et al., 2012, 2015; Christophel and Haynes, 2014), reported decoding of maintained information not only in early visual cortex but also in posterior parietal cortex. Further, decoding of stimulus position has been reported in both parietal and frontal cortex (Jerde et al., 2012; Sprague et al., 2014). While these results appear to disagree with the sensory recruitment model, they are potentially explained by considering the nature of the information that must be maintained and the underlying functional properties of the regions. Specifically, the novel stimuli employed by Christophel and colleagues are defined by the relative spatial position of the color or moving elements, precisely the kind of information that parietal cortex is generally thought to process during perception (Kravitz et al., 2011). Similarly, stimulus position is well represented in parietal and frontal cortex, related to sensory attention and motor behavior, making these regions a good substrate for maintaining representations of position in addition to early visual cortex. Taking into account that information may be maintained in brain regions more directly concerned with action, it has been suggested that “sensorimotor recruitment” rather than “sensory recruitment” may be a more appropriate way to think about maintained representations (D’Esposito and Postle, 2015).

Earlier we highlighted that the ability to maintain representations appears to be a general property of visual cortex. Given the evidence just discussed, it may be that this ability is not limited to visual cortex, but that any particular cortical region can be recruited for maintenance, depending on the nature of the information maintained. To test this idea, we presented participants sequentially with two visual objects before presenting a retro-cue (indicating which sample to hold in memory) and then asked them to perform one of two different tasks after a delay period (Lee et al., 2013). In the visual task participants were asked to indicate whether an object fragment presented after the delay belonged to the cued object or not, requiring the maintenance of visual features. In contrast, in the non-visual task, participants were asked to indicate whether a whole object presented after the delay was from the same subcategory or not, requiring the maintenance of the name or subcategory of the object. A separate behavioral experiment confirmed the nature of the information being maintained in the two tasks with visual object distractors presented in the delay period impairing performance on the visual-task more than the non-visual task and word distractors showing the opposite pattern. During the maintenance of visual properties, we found that object identity could be decoded from occipitotemporal but not prefrontal cortex. In contrast, during the maintenance of nonvisual properties (object category or name), we found that object identity could be decoded from prefrontal but not occipitotemporal cortex. These results confirm that information can be maintained in both prefrontal and visual cortex, but this maintenance is task-dependent and is stronger when the nature of the information matches the underlying functional properties of the region even for the same sample object. Further, the magnitude of activity in both regions was not modulated by task, providing further evidence that the magnitude of response during the delay period is dissociable from the presence or absence of maintained information.

One key prediction of the suggestion that information is maintained in regions that have functional properties matching the nature of that information is that there should be a correspondence between regions engaged during working memory and those engaged during perception of the same stimuli. For example, we suggested above that the decoding of maintained representations in posterior parietal cortex reported by Christophel et al. (2012, 2015) might reflect the complex visuospatial nature of their stimuli. We would therefore predict that those same regions should show strong decoding of the patterns during perception. Unfortunately, this was not tested in those studies. Similarly, it is unclear whether the parietal and frontal regions reported by Ester et al. (2015) also show decoding of orientation during perception.

More generally, it is possible that any region containing stimulus information during perception could maintain that information during working memory. In this context it is important to consider that, with sufficient power, stimulus-related responses for a simple visual stimulation plus attention control task are observed in the vast majority of the brain (Gonzalez-Castillo et al., 2012). If information can be widely distributed during perception, then the same may be true of maintenance during working memory. The failure to find more distributed maintained representations could reflect lack of power. As is always the case, the current null results should be treated very cautiously. In our own work, showing task-dependent decoding during the delay in occipitotemporal and prefrontal cortex (Lee et al., 2013), the critical result is the relative strength of decoding, not the presence or absence of decoding in either task.

Overall, multivoxel decoding studies have provided strong support for the role of visual cortex in the maintenance of information during visual working memory. However, the ability to maintain representations is not just limited to visual cortex and may be a general property of cortex with the nature of the information maintained determining which regions are engaged. In some cases (e.g., position, orientation), the information may be well represented in multiple regions and the decoding of maintained content may be highly distributed. In other cases (e.g., faces, objects) the information may be maintained only in regions with more specialized functional properties. Critically, the ability to maintain information is dissociable from the presence or absence of delay activity and elevated activity may reflect separate functions related to attention, motor preparation or executive control.

## Limitations of Multivoxel Decoding

Despite the advantages of decoding approaches for the study of maintenance during visual working memory, we need to be very cautious in interpreting the results (for discussion, see Serences and Saproo, 2012; Haynes, 2015).

First, although MVPA can provide evidence that there are distinct representations during visual working memory, it does not indicate what the nature of those representations are (Sligte et al., 2013). For example, Christophel and Haynes (2014) demonstrated decoding of maintained information about motion flowfields in MT+, posterior parietal cortex and somatosensory cortex. It is unlikely that the underlying neural representations are similar in these three areas, but all three areas show distinct responses to the different flowfields that may reflect different aspects of the stimuli or associated cognitive processing.

Second, the success of MVPA depends on the spatial arrangement of responses across voxels and may require the presence of large-scale maps (Freeman et al., 2011). Thus in V1, properties such as position and orientation can be readily decoded. The failure to find decoding for particular information in a given region could simply reflect heterogeneous organization of that information across the cortex rather than its absence.

Reconstruction of stimuli based on an underlying encoding model (Serences and Saproo, 2012) has the advantage of an explicit model of the underlying neural responses, making the presence of decoding more interpretable. Further, since the model is fit at the individual voxel level, the method is not dependent on the large-scale organization of information. However, this approach is dependent on the specific *a priori* assumptions made in generating the model. The assumption of orientation tuning is very reasonable for early visual cortex, but it is much more challenging to generate a model for higher cognitive functions.

## Relationship to Non-Human Primate Studies

In this section, we want to briefly discuss how the human multivoxel decoding results we have reviewed relate to findings in non-human primate literature, which have often focused on prefrontal cortex, and not visual cortex, as critical for the maintenance of information (for recent discussion, see also Postle, 2015b).

First, while there is strong evidence from the fMRI studies we have reviewed for maintained representations in early visual cortex (e.g., Harrison and Tong, 2009; Serences et al., 2009) and MT+ (e.g., Riggall and Postle, 2012), there is only limited evidence for maintained signals in non-human primate V1 (Supèr et al., 2001) and MT (Bisley et al., 2004; Zaksas and Pasternak, 2006). One account could be that these varying results reflect the very different nature of the signals recorded—single unit spiking data from non-human primates vs. population threshold and sub-threshold neural activity reflected in the BOLD response. Consistent with this view, a recent study found that the amplitude of local field potential (LFP) oscillations in macaque MT do reflect the maintained motion direction (Mendoza-Halliday et al., 2014). However, it is worth noting that that same study did find evidence for maintained representations of motion direction in firing rate in MST in addition to lateral prefrontal cortex (Mendoza-Halliday et al., 2014).

Second, while non-human primate studies have often reported stimulus-selective sustained activity in prefrontal cortex (e.g., Funahashi et al., 1989; Freedman et al., 2003), some fMRI decoding studies have failed to find evidence for maintained representations in human prefrontal cortex (e.g., Riggall and Postle, 2012; Emrich et al., 2013). Our emphasis on the nature of the maintained information could explain some of the discrepancy since the “cat” vs. “dog” category task employed by Freedman et al. (2003) may require abstract category information similar to that required in our non-visual task, which emphasized object name or category and revealed decoding in prefrontal cortex (Lee et al., 2013). However, as in posterior areas, the different nature of the signals measured with fMRI and neurophysiological recordings may also help explain the apparent discrepancies. Recent work has started to emphasize the dynamics of firing rate changes in monkey prefrontal cortex (Stokes, 2015) and a population level re-analysis of the data collected by Freedman and colleagues (Meyers et al., 2008) revealed a complex relationship over time between information in single neurons and that in the population as a whole. Further, neurophysiological recordings have revealed that a broad range of differ types of task features are reflected in the responses of prefrontal neurons (Stokes et al., 2013; Lara and Wallis, 2014; Postle, 2015b) and it may be difficult to tease these apart in the population-level measures reflected in the fMRI BOLD signals.

Finally, another potential account of the apparent discrepancy between the human and monkey studies is highlighted by a recent study of monkeys with unilateral prefrontal lesions (Pasternak et al., 2015). These monkeys exhibited a contralesional deficit in maintaining motion information across a delay, which was substantially pronounced when rapid allocation of spatial attention was required. This deficit was delay specific, supporting a role of prefrontal cortex in maintenance. Combined with the direction-selective signals recorded in prefrontal cortex during the delay period (Zaksas and Pasternak, 2006), this result might suggest a role for prefrontal cortex in maintaining the motion information necessary for this task. However, the deficit in the lesioned monkeys was not dependent on the specific stimulus features (coherence of the sample stimulus), suggesting it did not involve sensory information. Instead given the pronounced impact of rapidly shifting attention, the authors suggest that the role of prefrontal cortex lies in attending and accessing the task-relevant motion signals that are maintained elsewhere. Thus, the single unit neurophysiology data from non-human primate prefrontal cortex may be more associated with attentional signals than stimulus properties, while the multivoxel decoding data in human posterior cortex primarily reflects maintained sensory representations. Support for a specific role of prefrontal cortex in representing attentional context has also been provided by at least one multi-voxel decoding study (Nelissen et al., 2013).

## Relationship to Visual Mental Imagery

As we described earlier, the representation of information during visual working memory may be highly related to visual imagery. In both cases, visual information is represented in the absence of that information in the environment. The nature of the representations during visual imagery has been much debated (for review, see Pearson and Kosslyn, 2015). Recent evidence from multi-voxel decoding studies has provided strong support for the depictive (picture-like) view of visual imagery, which suggests visual imagery of a stimulus induces similar neural activation patterns with that generated by visual perception of the same stimulus (Stokes et al., 2009; Reddy et al., 2010; Cichy et al., 2012; Lee et al., 2012; Johnson and Johnson, 2014; Naselaris et al., 2015). For example, we trained participants to remember pictures of 10 common objects before placing them in the MRI scanner (Lee et al., 2012). During scanning, participants were cued with the name of the object and on interleaved trials were either presented with the picture of the object or asked to visually imagine the picture as vividly as possible. During imagery trials we found that we could decode the specific object the participant was imagining from responses in visual cortex. Furthermore, the patterns of response elicited during imagery were similar to those elicited during perception and it was possible to decode between imagery and perception suggesting that perception and imagery share similar substrates, much like the maintenance of information during visual working memory.

In comparing results from working memory with those from mental imagery it is worth noting that working memory paradigms involving a retro-cue, which requires the retrieval of previously presented information, are not that dissimilar from the paradigms used in mental imagery. The major difference is the time between presentation of the visual stimulation and the cue for retrieval.

To directly compare working memory and mental imagery, Albers et al. (2013) asked participants to perform two different tasks. In both cases, participants were first presented with a task cue followed by two serially presented gratings and then a second cue indicating which grating was relevant for that trial. In the working memory task, participants simply had to remember the cued grating over a delay period. Following the delay a probe stimulus was presented and participants indicated whether the probe was rotated clockwise or anticlockwise relative to the cued grating. In contrast, in the mental imagery task, participants had to mentally rotate the cued grating (with direction and angle indicated by the initial task cue) and then indicate whether the probe was rotated clockwise or anticlockwise relative to the imagined grating. Here the imagined grating is internally generated mental image that is novel but not remembered one. While Albers et al. (2013) refer to this as mental imagery, since the rotated image was never actually physically present, this task could also be interpreted as a short-term memory with manipulation task (i.e., requiring the working of “working memory”). They found that in V1-V3 they could decode orientation during the delay on both working memory and mental imagery trials. Furthermore, they could decode between tasks and there was also generalization to representations estimated during perception. These results suggest a common internal representation for visual working memory and mental imagery that is similar to that evoked during perception (Tong, 2013). Similar results were obtained by Christophel et al. (2015) with their color patterns, showing that transformed versions of the memorized stimulus could also be decoded from the same regions (early visual and posterior parietal cortex) as the original memorized stimulus.

In contrast to these results, Saad and Silvanto (2013) argued that working memory and visual imagery are partly dissociable processes. They asked participants to hold a grating in mind (visual short-term memory condition) or project it as a mental image on the computer screen (imagery condition), and compared the effect of each on visual perception. They found that both visual short-term memory (working memory) and imagery conditions were correlated with visual perception. However, while the subjective strength of visual imagery was negatively associated with visual perception, a positive correlation pattern was found for visual memory, suggesting dissociation. An alternative explanation for this is that the bottom-up visual input (screen), which is combined with the mental image (grating) in the imagery condition but not in the visual short-term memory condition, may interfere with visual stimuli for the visual perception performance. Thus, this dissociation may not reflect the different nature of signals for maintenance between imagery and working memory but interference effect between bottom-up visual inputs (Saad and Silvanto, 2013).

## Conclusion

In this article, we have reviewed fMRI studies employing multivoxel decoding during working memory. These studies have revealed maintained stimulus representations during delays that are unrelated to elevated activity levels. While these studies have often highlighted the role of early visual cortex, this may in part reflect the simple stimuli commonly employed and not any privileged role of early visual cortex in the process of maintenance. We have highlighted studies reporting decoding of maintained information outside of visual cortex and suggest that the distribution of representations during visual working memory is dependent on the information maintained, reflecting both the stimulus and the task. Thus, even prefrontal cortex may exhibit maintained representations for some types of information. Further, we suggest there should be correspondence between regions containing information during perception and those containing information during working memory and that any region that contains information during perception may potentially contribute to maintained representations during working memory. While we have focused on the maintenance of information, it is important to remember that there are many other aspects of working memory task performance that regions may contribute to, including stimulus-response mappings, match-nonmatch status of a trial, motor programs and decision criteria. Importantly we suggest that there may not be a sharp divide between regions involved in maintenance and regions involved in representing these aspects of task performance, but that these functions can co-exist in the same regions.

## Author Contributions

S-HL and CIB planned, discussed and wrote this article together.

## Funding

S-HL and CIB supported by the Intramural Research Program of NIMH (MH002909-08).

## Conflict of Interest Statement

The authors declare that the research was conducted in the absence of any commercial or financial relationships that could be construed as a potential conflict of interest.
